# The Effect of an AI-Based, Autonomous, Digital Health Intervention Using Precise Lifestyle Guidance on Blood Pressure in Adults With Hypertension: Single-Arm Nonrandomized Trial

**DOI:** 10.2196/51916

**Published:** 2024-05-28

**Authors:** Jared Leitner, Po-Han Chiang, Parag Agnihotri, Sujit Dey

**Affiliations:** 1 Electrical and Computer Engineering Department University of California, San Diego La Jolla, CA United States; 2 Department of Medicine University of California, San Diego La Jolla, CA United States

**Keywords:** blood pressure, hypertension, digital health, lifestyle change, lifestyle medicine, wearables, remote patient monitoring, artificial intelligence, AI, mobile phone

## Abstract

**Background:**

Home blood pressure (BP) monitoring with lifestyle coaching is effective in managing hypertension and reducing cardiovascular risk. However, traditional manual lifestyle coaching models significantly limit availability due to high operating costs and personnel requirements. Furthermore, the lack of patient lifestyle monitoring and clinician time constraints can prevent personalized coaching on lifestyle modifications.

**Objective:**

This study assesses the effectiveness of a fully digital, autonomous, and artificial intelligence (AI)–based lifestyle coaching program on achieving BP control among adults with hypertension.

**Methods:**

Participants were enrolled in a single-arm nonrandomized trial in which they received a BP monitor and wearable activity tracker. Data were collected from these devices and a questionnaire mobile app, which were used to train personalized machine learning models that enabled precision lifestyle coaching delivered to participants via SMS text messaging and a mobile app. The primary outcomes included (1) the changes in systolic and diastolic BP from baseline to 12 and 24 weeks and (2) the percentage change of participants in the controlled, stage-1, and stage-2 hypertension categories from baseline to 12 and 24 weeks. Secondary outcomes included (1) the participant engagement rate as measured by data collection consistency and (2) the number of manual clinician outreaches.

**Results:**

In total, 141 participants were monitored over 24 weeks. At 12 weeks, systolic and diastolic BP decreased by 5.6 mm Hg (95% CI −7.1 to −4.2; *P*<.001) and 3.8 mm Hg (95% CI −4.7 to −2.8; *P*<.001), respectively. Particularly, for participants starting with stage-2 hypertension, systolic and diastolic BP decreased by 9.6 mm Hg (95% CI −12.2 to −6.9; *P*<.001) and 5.7 mm Hg (95% CI −7.6 to −3.9; *P*<.001), respectively. At 24 weeks, systolic and diastolic BP decreased by 8.1 mm Hg (95% CI −10.1 to −6.1; *P*<.001) and 5.1 mm Hg (95% CI −6.2 to −3.9; *P*<.001), respectively. For participants starting with stage-2 hypertension, systolic and diastolic BP decreased by 14.2 mm Hg (95% CI −17.7 to −10.7; *P*<.001) and 8.1 mm Hg (95% CI −10.4 to −5.7; *P*<.001), respectively, at 24 weeks. The percentage of participants with controlled BP increased by 17.2% (22/128; *P*<.001) and 26.5% (27/102; *P*<.001) from baseline to 12 and 24 weeks, respectively. The percentage of participants with stage-2 hypertension decreased by 25% (32/128; *P*<.001) and 26.5% (27/102; *P*<.001) from baseline to 12 and 24 weeks, respectively. The average weekly participant engagement rate was 92% (SD 3.9%), and only 5.9% (6/102) of the participants required manual outreach over 24 weeks.

**Conclusions:**

The study demonstrates the potential of fully digital, autonomous, and AI-based lifestyle coaching to achieve meaningful BP improvements and high engagement for patients with hypertension while substantially reducing clinician workloads.

**Trial Registration:**

ClinicalTrials.gov NCT06337734; https://clinicaltrials.gov/study/NCT06337734

## Introduction

### Background

High blood pressure (BP), or hypertension, is one of the most prevalent chronic diseases in the world [1]. Hypertension affects 48% (approximately 120 million) of adults in the United States, and 78% (approximately 93 million) of the cases are uncontrolled (ie, BP≥130/80 mm Hg) [2]. Hypertension is a major risk factor for stroke and acute myocardial infarction [3] and remains a large public health challenge with an extra cost of US $2000 per year per hypertension patient, resulting in an additional US $131 billion in annual health care costs in the United States [4]. The American College of Cardiology and American Heart Association’s clinical practice guidelines define hypertension as systolic BP (SBP)≥130 mm Hg or diastolic BP (DBP)≥80 mm Hg, consistently over time [5]. A large-scale analysis of 48 randomized clinical trials showed that a 5–mm Hg reduction in SBP lowered the risk of major cardiovascular events by 10% [6], highlighting the importance of developing new strategies to achieve hypertension control at scale.

Hypertension management typically begins with home monitoring of BP to gain a more accurate estimate of a patient’s BP within their usual, daily routine [7]. However, self-monitoring without additional support is not associated with lower BP or better control [8-10]. Lifestyle management in conjunction with self-monitoring is effective in controlling BP as lifestyle factors (eg, activity, sleep, diet, and stress) have a substantial impact on BP [11-14]. Even for patients taking antihypertensive medication, lifestyle management can enhance medication efficacy, leading to better BP control [15]. Traditionally, lifestyle management involves patients with hypertension visiting their primary care physician (PCP) and receiving guidance on lifestyle modifications that are generally known to improve BP. However, due to time constraints related to workload, physicians are often unable to optimally counsel patients on lifestyle modifications or personalize their guidance [16,17]. Due to insufficient guidance and the lack of feedback in between clinic visits, patients may implement some of these changes; however, patient engagement and compliance are generally suboptimal for achieving control. To improve patient engagement, new digital health technologies and remote patient monitoring programs have been developed for hypertension care [18-21]. These programs typically provide patients with remote monitoring devices (eg, BP cuffs and activity trackers) and match patients with health coaches. BP and lifestyle data collected from remote monitoring devices allow health coaches to view trends and make personalized recommendations to patients. However, these approaches do not consider the individual impact of lifestyle factors on BP, which may vary across individuals due to physiological differences. Furthermore, the reliance on health coaches is highly time and resource intensive, resulting in a high operating cost, which significantly limits scalability [22].

### Objectives

To address the challenges of poor patient engagement due to generic, insufficient guidance and limited scalability of care due to human coaching models, we propose an artificial intelligence (AI)–driven, autonomous, precise lifestyle coaching program for patients with hypertension. The intervention platform consists of a monitoring system that ingests lifestyle and BP data and builds personalized machine learning (ML) models to determine the individual impact of different lifestyle factors on BP. On the basis of the lifestyle impact analysis, the system autonomously provides precise lifestyle recommendations delivered to a patient’s smartphone that enable patients to focus on specific aspects of their lifestyle that have the greatest associations with their BP. While the platform autonomously engages patients, it is clinician supervised and notifies clinicians of critical BP readings. In our previous study [23], we enrolled 38 participants who were prehypertensive or had stage-1 hypertension (SBP between 120 and 139 mm Hg or DBP between 80 and 89 mm Hg) and demonstrated that 75% of the participants receiving the intervention were able to achieve a controlled BP (<130/80 mm Hg) after 16 weeks of engagement. However, the limitations of the previous study [23] are as follows: (1) the participants were not provided with an interactive mobile app for the delivery of our precise lifestyle recommendations, (2) the small number of participants did not enable rigorous evaluation, and (3) the study did not consider patients with stage-2 hypertension who can potentially benefit more from lifestyle management.

This study aims to evaluate the effectiveness of our AI-based, precise lifestyle guidance coaching program in helping patients with stage-2 hypertension achieve BP control and demonstrate the platform’s scalability. The primary study objectives are to evaluate the change in BP and the percentage change of participants in different BP categories (controlled, stage-1 hypertension, and stage-2 hypertension) over time (baseline, 12 weeks, and 24 weeks). Secondary objectives include assessing participant engagement as measured by consistency of data collection and interactions with our mobile app and determining the number of manual clinician interventions, as defined by the escalation rules set for the study, to assess the potential scalability of our approach.

## Methods

### Recruitment

This study was performed in collaboration with the University of California, San Diego Health’s Population Health Services Organization (PHSO). Participants were enrolled on a rolling basis from November 2021 to February 2023. The inclusion criteria required participants to have stage-2 hypertension (SBP≥140 mm Hg or DBP≥90 mm Hg per the American College of Cardiology and American Heart Association’s 2017 guidelines [5]) based on their most recent clinical measurements and to be fully ambulatory (ie, not requiring an assistive device such as a cane, wheelchair, or walker). In addition, participants were required to be aged ≥18 years at enrollment, be English speaking, and own an Android or iPhone (Apple Inc) smartphone. The trial was designed in a fully remote manner so that participants could participate entirely from home. The PHSO care team aggregated a list of patients who met the inclusion criteria and sent a recruitment flyer via bulk message using the Epic MyChart (Epic Systems Corporation) messenger. The flyer introduced the study and instructed patients to email the study team if they were interested in participating. After contacting the study team, eligible patients were asked to complete an electronic informed consent form. Patients who consented were sent a Fitbit Inspire 2 (Fitbit Inc) and a Bluetooth-enabled Omron Silver (Omron Corporation) BP monitor to collect their lifestyle and BP data for up to 6 months. Each shipment included instructions for self-onboarding, which described the steps to set up and connect the devices to the patient’s mobile phone. Patients who already owned a Fitbit or Apple Watch (Apple Inc) had the option to use their device instead of receiving one from the study team. Patients who required an extra-large cuff were provided an iHealth Ease (iHealth Labs Inc) BP monitor instead of an Omron Silver.

### Ethical Considerations

This study (protocol #181405) was reviewed and approved by the University of California, San Diego’s Human Research Protections Program, which operates Institutional Review Boards. All participants in this study provided informed consent, which included the collection of their data and the provision of study results derived from their individual data. The confidentiality and privacy of participants were ensured by assigning a deidentified code to each patient. While participants were not offered monetary compensation, those without a BP monitor or wearable device were provided with these devices. The study was registered at ClinicalTrials.gov (NCT06337734).

### Study Design and Data Collection

**Figure 1:**
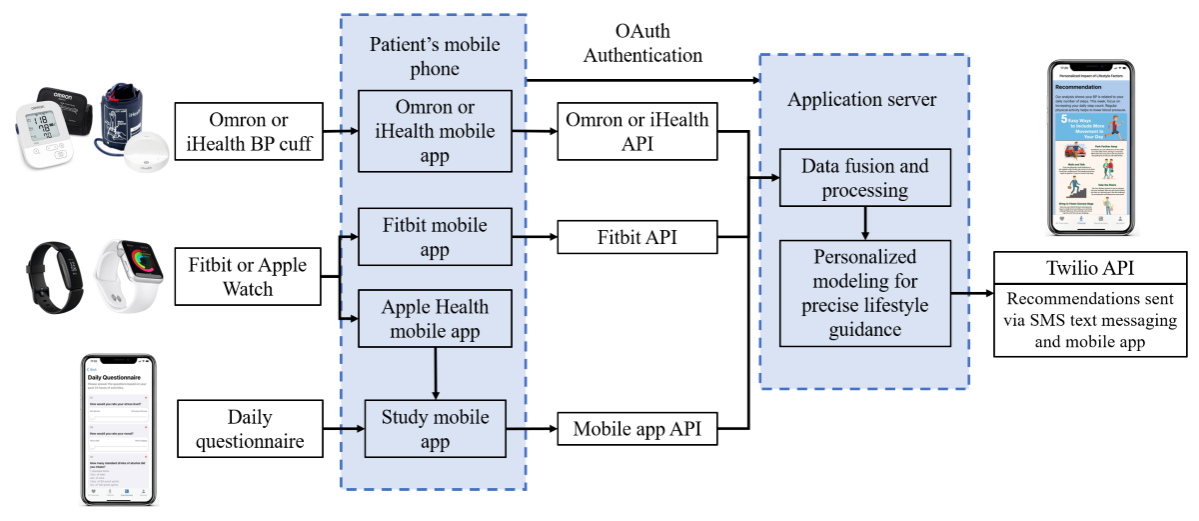
Architecture of data transmission. Participant data were collected from Bluetooth-enabled blood pressure (BP) monitors, wearable devices, and a mobile app–based questionnaire. Data were uploaded through the respective application programming interfaces (APIs) to our app server, where the individualized analysis was carried out before delivering recommendations to participants.

We collected data from each participant using a Fitbit or Apple Watch, Omron or iHealth wireless BP monitor, and the study’s questionnaire mobile app. Participants were asked to wear their Fitbit or Apple Watch as often as possible, including during sleep, and take 1 to 2 BP measurements per day, in the morning (8 AM-10 AM) or evening (7 PM-9 PM). We provided participants with instructions on how to take accurate resting BP readings [24] and asked that they take 3 consecutive readings during each morning and evening session. This resulted in 1 to 2 sets of 3 measurements per day, and the average of the 3 measurements was used as the final value for each session. Participants synced their BP data to the Omron or iHealth mobile app and their Fitbit data to the Fitbit mobile app; subsequently, the data were automatically uploaded to the Omron, iHealth, or Fitbit clouds. These data were retrieved remotely through the application programming interfaces (APIs) provided by Omron, iHealth, and Fitbit. Data from the Apple Watch were synced with the study mobile app and uploaded via a custom API to our server. In addition, participants completed a daily questionnaire using our study mobile app that asked about their stress, mood, and dietary choices over the past 24 hours. These questions were developed in collaboration with physicians on our team. The diet questions are tailored to measure information relevant to hypertension, including alcohol, red meat, fruits or vegetables, and salt consumption [25]. The details of the questionnaire are described in our previous study [23]. In addition, we asked participants to complete a study experience survey that asked them to rate the difficulty level of completing the study tasks, how useful they found the recommendations, and their experience using the app. These responses were collected through the mobile app and used to assess participant experience. Figure 1 describes the system architecture and data transmission.

Wrist-worn activity and sleep trackers have been widely used in health-related research studies [26], and devices such as Fitbits and Apple Watches have been shown to accurately measure parameters such as step count, heart rate, and sleep duration [27,28]. Fitbits and Apple Watches include an optical heart rate monitor and a 3-axis accelerometer. The devices use these sensors to calculate various health parameters, including lifestyle and vitals measurements. Lifestyle factors include activity (eg, steps, walking and running speed, and active time), sleep timing (eg, sleep duration, bedtime, and uptime), and sleep stages (ie, deep, light, rapid eye movement, and awake). These lifestyle factors are used as part of the intervention, in which we use ML techniques to determine which of the factors have the greatest association with a participant’s BP and base our guidance on this analysis.

### Description of the Intervention

The intervention is intended to support participants’ daily efforts to improve BP and overall cardiometabolic function by facilitating behavioral changes that target physical activity, sleep hygiene, stress management, and dietary choices most relevant to their BP. The intervention platform uses remotely collected lifestyle and BP data to provide personalized, precise, and proactive lifestyle coaching using AI to participants with hypertension. The system integrates the data described in the previous section into a combined data set for each participant. Each participant’s personal data set consists of lifestyle features (eg, step count, sleep duration, and salt consumption) that are time aligned with their BP measurements, which serve as the labels for training the ML model. Therefore, each participant’s data set is used to train a personal ML model that can predict BP using the participant’s lifestyle data as input. With this trained model, the intervention system can determine how different aspects of lifestyle affect the participant’s BP. On the basis of the model’s determination of the lifestyle factors’ impact, the system generates precise lifestyle recommendations. Each lifestyle factor is mapped to a corresponding lifestyle recommendation that was designed with physicians on our team to be consistent with evidence-based clinical guidelines. Furthermore, prior studies have demonstrated that these recommendations, such as increasing step count [29,30], improving sleep quality [31,32], managing stress [33], and reducing salt consumption [34,35], can result in BP reduction. The objective of these precise lifestyle recommendations is to encourage participants to concentrate on 1 aspect of their lifestyle at a time, focusing on the factor that has the greatest association with their BP based on the underlying relationship between their BP and lifestyle factors. We describe the AI-based intervention platform in more detail in our previous study [23].

**Figure 2:**
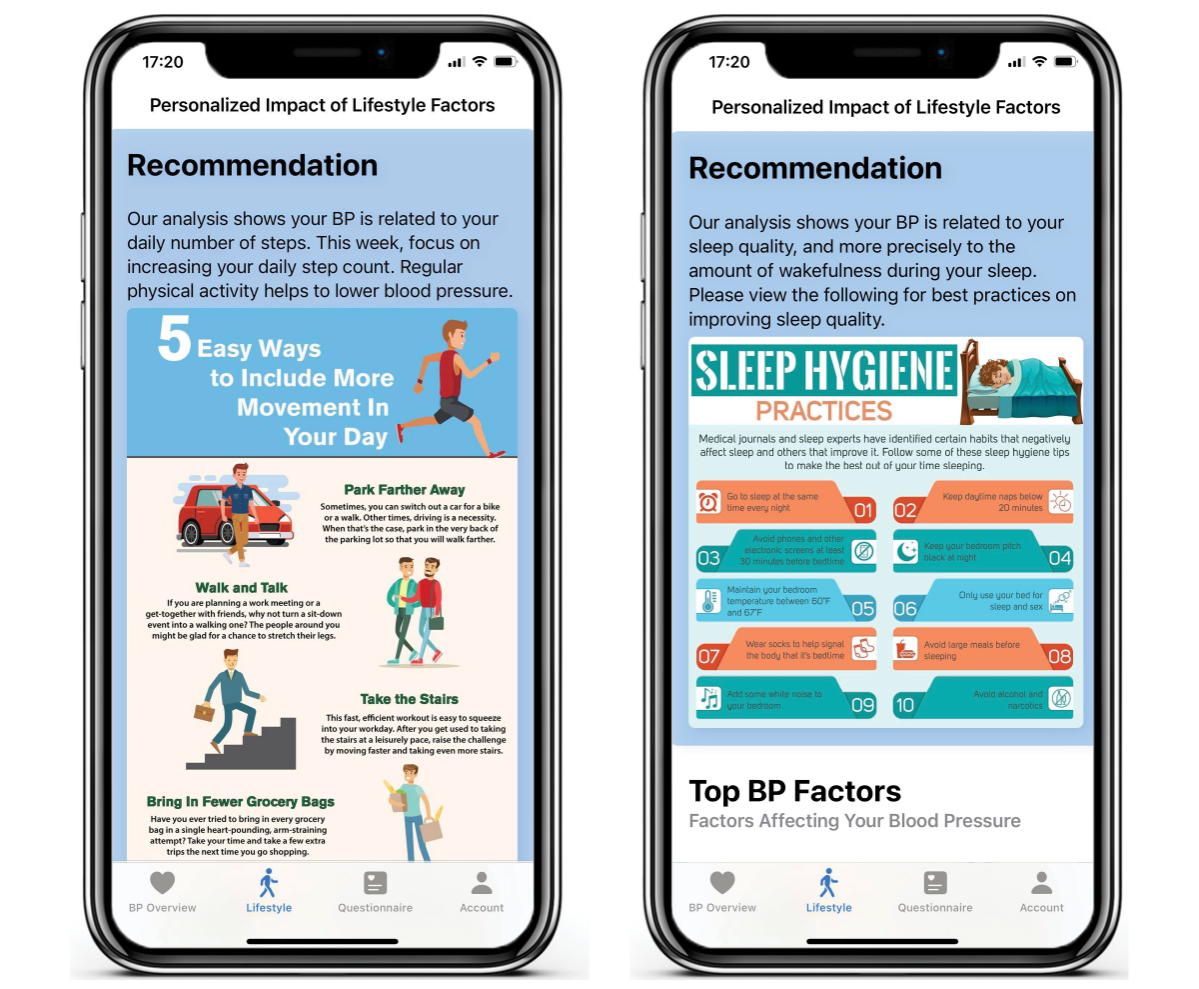
Lifestyle recommendations delivered in the mobile app. Participants received weekly lifestyle recommendations based on their data and personalized analytics. The recommendations encouraged participants to prioritize a single lifestyle modification at a time, focusing on the factor that had the greatest impact on their blood pressure (BP).

Participants received weekly lifestyle recommendations based on their data and personalized analytics, which continuously evolved over time. These recommendations were delivered to participants via programmable text messages using the Twilio API (Twilio Inc) service [36] and were displayed in the study mobile app. Each text message included a summary of the participant’s BP progression for the current week in addition to the lifestyle recommendation. Figure 2 displays examples of these weekly lifestyle recommendations provided in the study app. In addition, patients completed a midweek check-in on the app, which asked whether they could follow each recommendation (yes or no) and to rate the recommendation difficulty on a scale from 1 to 5.

The system includes a safety mechanism to involve clinician intervention in the case of critically high or low BP readings. Critically high BP was defined as SBP>180 mm Hg or DBP>110 mm Hg, and critically low BP was defined as SBP<90 mm Hg or DBP<60 mm Hg [5]. After a critical reading, participants received a text message asking them to remeasure their BP and prompting them to seek assistance or call their medical provider if they were experiencing certain symptoms (eg, chest pain and severe headache). After 2 critical readings in a row, an escalation notification was sent to the PHSO care team via email for manual outreach. To avoid notification fatigue, we limited the number of critically high or low BP notifications sent to the care team to 1 notification per week for a patient.

### Primary Outcomes: BP Change and Population Hypertension Control

The first primary outcome was the change in SBP and DBP from baseline to 12 weeks and 24 weeks. A participant’s baseline BP was calculated as the average of their readings during the first week of the study. The 12th- and 24th-week BPs were a participant’s average reading during that week of the study plus 1 week and minus 1 week. We included BP measurements from 1 week before and after to get a more representative result. For example, the 12-week value was the average of all readings from weeks 11 to 13. As previously mentioned, a 5–mm Hg reduction in SBP can lower the risk of major cardiovascular events by 10% [6]. This motivated us to determine the percentage of participants who experienced >5–mm Hg reduction in SBP at 12 weeks and 24 weeks. To understand the effect on participants with different baseline BPs, we carried out subgroup analysis in which participants were sorted into 3 groups based on their baseline BP: (1) controlled (SBP<130 mm Hg and DBP<80 mm Hg), (2) stage-1 hypertension (SBP 130-139 mm Hg or DBP 80-89 mm Hg), and (3) stage-2 hypertension (SBP≥140 mm Hg or DBP≥90 mm Hg).

Another primary outcome was the percentage change of participants in different BP categories from baseline to 12 weeks and 24 weeks. To assess this, we calculated the percentage of participants who were in the controlled, stage-1 hypertension, and stage-2 hypertension categories at baseline, 12 weeks, and 24 weeks. Using these percentages, we determined the percentage change from baseline to 12 weeks and 24 weeks.

### Secondary Outcomes: Participant Engagement and Clinician Intervention

A secondary outcome measured participant engagement as determined by the consistency of data collection and interactions with our mobile app. The 3 main tasks participants were asked to complete included measuring BP, syncing their wearable device, and answering the mobile app questionnaire. As a result, we used these 3 tasks as our measure of engagement and calculated the percentage of participants completing each of these tasks each week. A participant was marked as engaged for a given week if they provided a BP reading, synced their wearable device data, and answered the questionnaire at least once during the week.

Another secondary outcome was the number of times participants were escalated to the PHSO care team for manual follow-up. The objective of this outcome was to determine the care team’s time and resource requirements to implement the intervention and assess the scalability of our approach. The condition for care team intervention was 2 critical BP readings in a row, as previously described.

### Statistical Analysis

Descriptive statistics (eg, mean, SD, and percentage) were calculated to describe the demographic and baseline clinical characteristics of the enrolled study population. We compared the characteristics between subgroups based on their baseline BP classification.

Change in SBP and DBP from baseline to 12 weeks and 24 weeks was analyzed using a 2-tailed paired Student *t* test with the level of statistical significance set to *P*<.05. Furthermore, 95% CIs were calculated for these changes. Baseline and follow-up BP data were normally distributed. The McNemar nonparametric test was used to examine the change in the proportion of participants in the controlled, stage-1, and stage-2 BP range from baseline to 12 weeks and 24 weeks. The McNemar test is used to determine if there is a statistically significant difference in proportions between paired data. We conducted all statistical analyses with Python 3.9 (Python Software Foundation) using the *NumPy*, *Pandas*, and *SciPy* libraries.

## Results

### Feasibility Outcomes: Recruitment, Adherence, and Participant Experience

**Figure 3:**
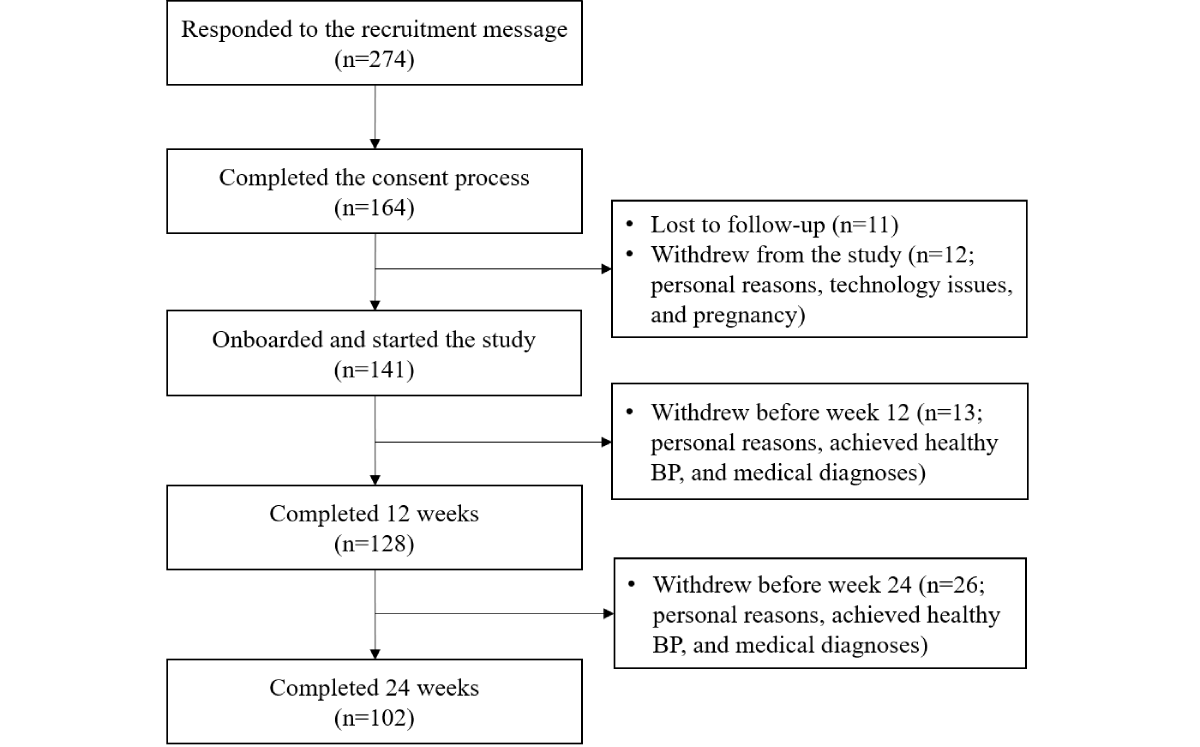
Flow of participants through the study. Adults with hypertension were enrolled from the University of California, San Diego Health between November 2021 and February 2023 into a single-arm nonrandomized trial. BP: blood pressure.

Participants were enrolled on a rolling basis from November 2021 to February 2023. Figure 3 details the recruitment numbers and participant flow through the study. A total of 274 patients responded to the Epic MyChart recruitment message by contacting our team and expressing interest. In total, 164 patients consented to join the study, out of which 141 (86%) were onboarded and started collecting data. There was a 9.2% (13/141) dropout rate from the start of the study to 12 weeks and a 20.3% (26/128) dropout rate from 12 weeks to 24 weeks. Reasons for participants withdrawing from the study included receiving new medical diagnoses (eg, cancer diagnosis), achieving a healthy BP, family emergencies, and other personal reasons. For the 141 participants who onboarded, Table 1 compares the characteristics between subgroups based on baseline BP classifications. The average age of participants was 57.5 (SD 13.9) years, and 44% (62/141) of the participants were female. For participants who had stage 2 hypertension at baseline, the average baseline BP was 141.9/89.4 mm Hg. In total, 83.7% (118/141) of the participants reported that they were taking antihypertensive medication at the beginning of the study.

**Figure 4:**
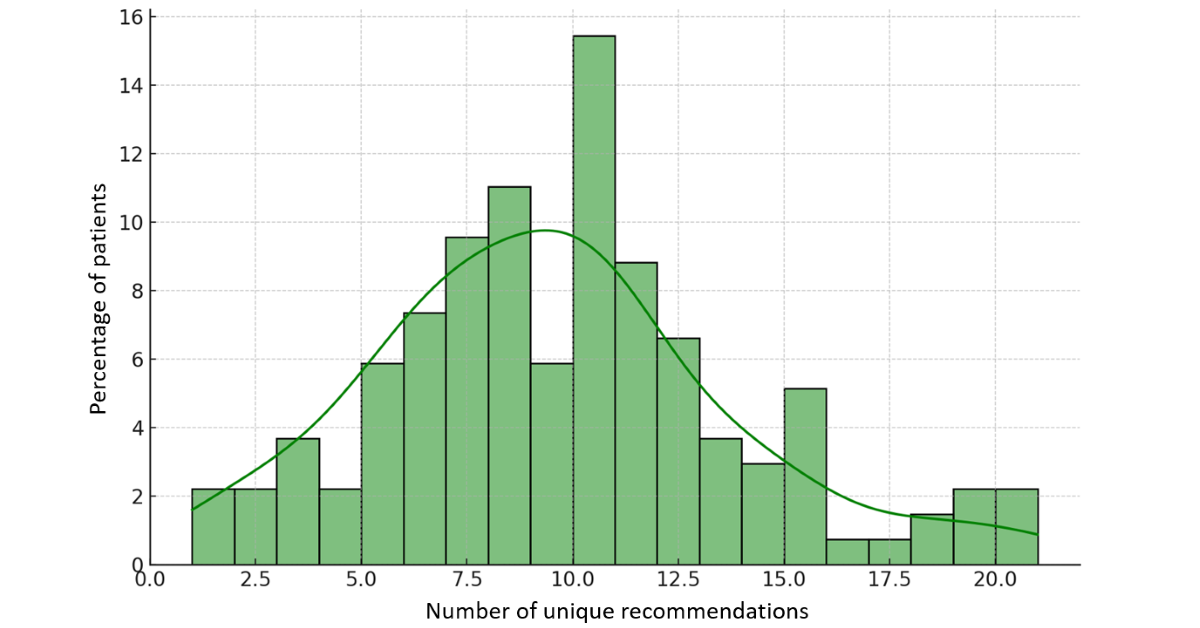
Distribution showing the number of unique recommendations sent to each patient. Patients received an average of 9.4 unique recommendations each.

As previously described, we asked participants each week to rate the difficulty of the recommendations they received on a scale from 1 to 5 and indicate whether they could follow each recommendation. This was done to assess compliance and the perceived difficulty of the recommendations. The histogram of difficulty ratings, divided into *Yes* and *No* responses, is shown in Multimedia Appendix 1. Recommendations were followed 63.64% (721/1133) of the time and not followed 36.36% (412/1133) of the time. The average difficulty rating for recommendations that were followed was 1.97, indicating lower difficulty, whereas the average for those not followed was 3.67, indicating higher difficulty. Evidently, there is a negative correlation between the perceived difficulty of a recommendation and its likelihood of being followed. We also tracked the number of unique recommendations each patient was sent. Out of the 37 unique recommendations, patients received an average of 9.4 (25%) unique recommendations each. The distribution of the number of unique recommendations is shown in Figure 4. The median and IQR suggest a distribution close to normal. The maximum number of unique recommendations received by a single patient was as high as 21. These statistics demonstrate a broad range of recommendations given to the patients, covering various aspects of lifestyle.

An additional feasibility outcome we evaluated was participant experience as measured by responses to a study experience survey. As previously mentioned, this survey asked patients to rate the difficulty level of completing the study tasks, how useful they found the recommendations, and their experience using the app. Multimedia Appendix 2 presents the distribution of participant responses to these 3 questions. In total, 70 participants responded to the survey. In total, 61% (43/70) of the participants responded that the study tasks were “easy” or “very easy” to incorporate into their daily routine, 51% (36/70) of the participants found the personalized recommendations to be “useful” or “very useful” compared to generic recommendations, and 86% (60/70) of the participants rated the app experience as “good” or “great.”

**Table 1:** Participant demographics and characteristics grouped by baseline BP^a^ (N=141).

Characteristics	Baseline BP category			
	All (N=141)	Controlled (n=38)	Stage 1 (n=48)	Stage 2 (n=55)
Age (y), mean (SD)	57.5 (13.9)	57.8 (16.0)	57.6 (12.6)	57.3 (13.5)
Female, n (%)	62 (44)	14 (37)	24 (50)	24 (44)
Weight (lb), mean (SD)	175.8 (48.4)	170.0 (41.6)	164.5 (52.3)	189.7 (45.7)
Baseline SBP^b^ (mm Hg), mean (SD)	131.9 (11.5)	121.4 (6.1)	128.8 (7.1)	141.9 (9.3)
Baseline DBP^c^ (mm Hg), mean (SD)	82.9 (9.0)	74.2 (4.4)	82.2 (6.4)	89.4 (8.0)
Taking hypertension medication, n (%)	118 (83.7)	32 (84)	39 (81)	47 (85)

^a^BP: blood pressure.

^b^SBP: systolic blood pressure.

^c^DBP: diastolic blood pressure.

### BP Outcomes

For assessing BP outcomes, we used data from the 128 and 102 participants who completed 12 and 24 weeks in the study, respectively. Table 2 details the change in BP from baseline to 12 weeks. Across all participants, there was a statistically significant change of −5.6 mm Hg (95% CI −7.1 to −4.2; *t_127_*=7.6; *P*<.001) in SBP and −3.8 mm Hg (95% CI −4.7 to −2.8; *t_127_*=7.7; *P*<.001) in DBP after 12 weeks. Notably, 45.3% (58/128) of the participants achieved a clinically meaningful SBP drop of ≥5 mm Hg after 12 weeks. Table 3 details the change in BP from baseline to 24 weeks. For the participants who completed 24 weeks in the study, there was a statistically significant change of −8.1 mm Hg (95% CI −10.1 to −6.1; t_101_=8.1; *P*<.001) in SBP and −5.1 mm Hg (95% CI −6.2 to −3.9; t_101_=8.4; *P*<.001) in DBP. In total, 58.8% (60/102) of the participants achieved a clinically meaningful SBP drop of ≥5 mm Hg after 24 weeks.

**Table 2:** Comparison of average BP^a^ change at 12 weeks for different participant subgroups based on baseline BP (n=128)^b^.

BP and subgroup		Participants, n (%)	Change in BP at 12 weeks, Δmean (SD; 95% CI)	*t* test (*df*)	*P* value	≥5–mm Hg reduction in SBP^c^ at 12 weeks, n (%)
**SBP**						
	Overall	128 (100)	−5.6 (8.1; −7.1 to −4.2)	7.6 (127)	<.001	58 (45.3)
	Controlled	31 (24.2)	−3.6 (5.2; −5.5 to −1.6)	3.7 (30)	.001	11 (35)
	Stage 1	46 (35.9)	−2.6 (7.2; −4.8 to −0.5)	2.5 (45)	.02	14 (30)
	Stage 2	51 (39.8)	−9.6 (9.2; −12.2 to −6.9)	7.3 (50)	<.001	33 (65)
**DBP^d^**						
	Overall	128 (100)	−3.8 (5.5; −4.7 to −2.8)	7.7 (127)	<.001	N/A^e^
	Controlled	31 (24.2)	−1.6 (3.8; −3.0 to −0.2)	2.3 (30)	.03	N/A
	Stage 1	46 (35.9)	−3.1 (4.4; −4.4 to −1.7)	4.7 (45)	<.001	N/A
	Stage 2	51 (39.8)	−5.7 (6.7; −7.6 to −3.9)	6.2 (50)	<.001	N/A

^a^BP: blood pressure.

^b^For participants with stage-2 hypertension at baseline, SBP and DBP changed by −9.6 mm Hg and −5.7 mm Hg, respectively, after 12 weeks.

^c^SBP: systolic blood pressure.

^d^DBP: diastolic blood pressure.

^e^N/A: not applicable.

**Table 3:** Comparison of average BP^a^ change at 24 weeks for different participant subgroups based on baseline BP (n=102)^b^.

BP and subgroups			Participants, n (%)		Change in BP at 24 weeks, Δmean (SD; 95% CI)		*t* test (*df*)		*P* value		≥5–mm Hg reduction in SBP^c^ at 24 weeks, n (%)
**SBP**											
	Overall	102 (100)		−8.1 (10.1; −10.1 to −6.1)		8.1 (101)		<.001		60 (58.8)	
	Controlled	28 (27.5)		−3.9 (8.6; −7.1 to −0.8)		2.6 (27)		.02		14 (50)	
	Stage 1	37 (36.3)		−5.2 (8.0; −7.9 to −2.5)		3.9 (36)		<.001		17 (46)	
	Stage 2	37 (36.3)		−14.2 (10.6; −17.7 to −10.7)		8.2 (36)		<.001		29 (78)	
**DBP^d^**											
	Overall	102 (100)		−5.1 (6.0; −6.2 to −3.9)		8.4 (101)		<.001		N/A^e^	
	Controlled	28 (27.5)		−1.9 (4.3; −3.6 to −0.2)		2.3 (27)		.03		N/A	
	Stage 1	37 (36.3)		−4.4 (4.7; −6.0 to −2.8)		5.7 (36)		<.001		N/A	
	Stage 2	37 (36.3)		−8.1 (6.9; −10.4 to −5.7)		7.0 (36)		<.001		N/A	

^a^BP: blood pressure.

^b^For participants with stage-2 hypertension at baseline, SBP and DBP changed by −14.2 mm Hg and −8.1 mm Hg, respectively, after 24 weeks.

^c^SBP: systolic blood pressure.

^d^DBP: diastolic blood pressure.

^e^N/A: not applicable.

Participants with a baseline BP classified as stage-2 hypertension had the greatest change in BP and the greatest percentage of participants achieving a clinically meaningful SBP drop after 12 and 24 weeks. For these participants, SBP and DBP improved by −9.6 mm Hg (95% CI −12.2 to −6.9; t_50_=7.3; *P*<.001) and −5.7 mm Hg (95% CI −7.6 to −3.9; t_50_=6.2; *P*<.001) after 12 weeks, respectively, and −14.2 mm Hg (95% CI −17.7 to −10.7; t_36_=8.2; *P*<.001) and −8.1 mm Hg (95% CI −10.4 to −5.7; t_36_=7.0; *P*<.001) after 24 weeks, respectively. In total, 65% (33/51) and 78% (29/37) of the participants achieved a clinically meaningful SBP drop of ≥5 mm Hg after 12 and 24 weeks, respectively.

Another primary outcome we assessed was the percentage change of participants in different BP categories from baseline to 12 weeks and 24 weeks. Tables 4 and 5 detail this analysis. For participants completing 12 weeks in the study, the percentage of participants in the controlled range increased by 17.2% from 24.2% (31/128) to 41.4% (53/128; McNemar *χ*^2^_1_=3.0, *P*<.001). The percentage of participants with stage 2 hypertension decreased by 25% from 39.8% (51/128) to 14.8% (19/128; McNemar *χ*^2^_1_=4.0, *P*<.001) after 12 weeks. This means that 63% (32/51) of the patients with stage-2 hypertension at baseline moved into lower BP categories after 12 weeks. For those who completed 24 weeks in the study, the percentage in the controlled range increased by 26.5% from 27.5% (28/102) to 53.9% (55/102; McNemar *χ*^2^_1_=2.0, *P*<.001), and the stage-2 percentage decreased by 26.5% from 36.3% (37/102) to 9.8% (10/102; McNemar *χ*^2^_1_=3.0, *P*<.001). This means that 73% (27/37) of the patients with stage-2 hypertension at baseline moved into lower BP categories after 24 weeks. Note that the percentage changes for the stage-1 hypertension category from baseline to 12 weeks and 24 weeks were not statistically significant at the *P*=.05 level. The smaller change in the stage-1 hypertension population is due to a cascading effect where the number of participants moving from stage 2 into stage 1 was offset by the number of patients moving out of stage 1 and into the controlled BP category. For example, from baseline to 24 weeks, 18 participants moved from stage 2 to stage 1, and 17 participants moved from stage 1 to the controlled category.

**Table 4:** Change in the percentage of participants in different BP^a^ categories from baseline to 12 weeks (n=128)^b^.

Subgroups	Population at baseline, n (%)	Population at 12 weeks, n (%)	12-week difference, n (%)	McNemar *χ*^2^ (*df*)	*P* value
Controlled	31 (24.2)	53 (41.4)	22 (17.2)	3.0 (1)	<.001
Stage 1	46 (35.9)	56 (43.8)	10 (7.8)	20.0 (1)	.20
Stage 2	51 (39.8)	19 (14.8)	−32 (−25)	4.0 (1)	<.001

^a^BP: blood pressure.

^b^The percentage of participants with stage-2 hypertension decreased by 25% from 39.8% to 14.8% after 12 weeks.

**Table 5:** Change in the percentage of participants in different BP^a^ categories from baseline to 24 weeks (n=102)^b^.

Subgroups	Population at baseline, n (%)	Population at 24 weeks, n (%)	24-week difference, n (%)	McNemar *χ*^2^ (*df*)	*P* value
Controlled	28 (27.5)	55 (53.9)	27 (26.5)	2.0 (1)	<.001
Stage 1	37 (36.3)	37 (36.3)	0 (0)	N/A^c^	N/A
Stage 2	37 (36.3)	10 (9.8)	−27 (−26.5)	3.0 (1)	<.001

^a^BP: blood pressure.

^b^The percentage of participants with stage-2 hypertension decreased by 26.5% from 36.3% to 9.8% after 24 weeks.

^c^N/A: not applicable.

### Participant Engagement

**Figure 5:**
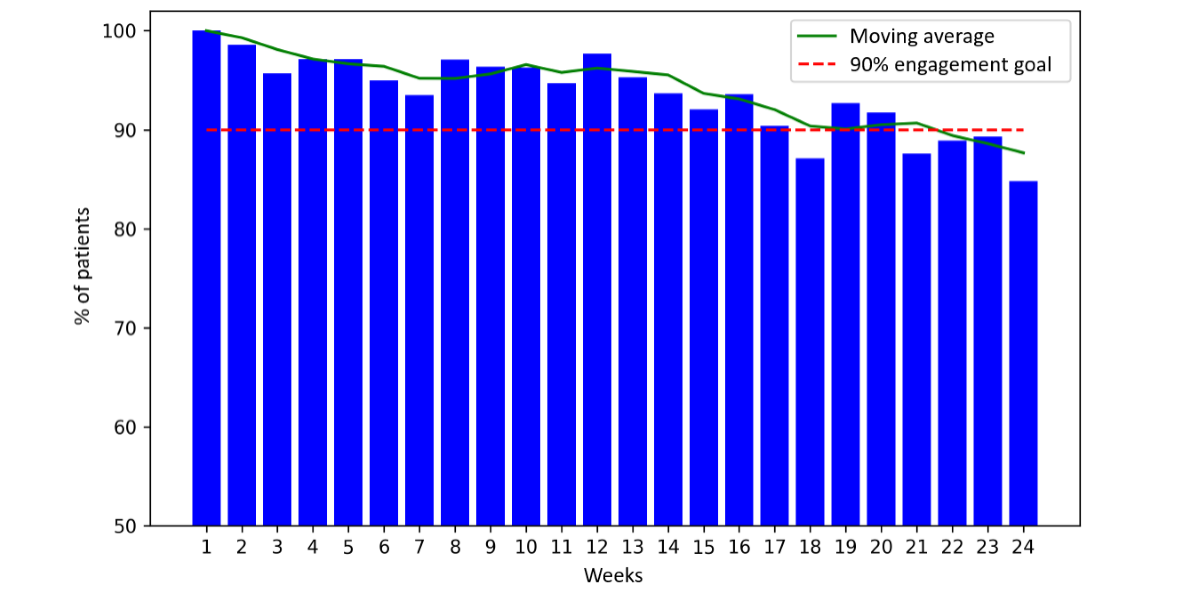
Percentage of active participants measuring their blood pressure (BP) during the 24 weeks.

**Figure 6:**
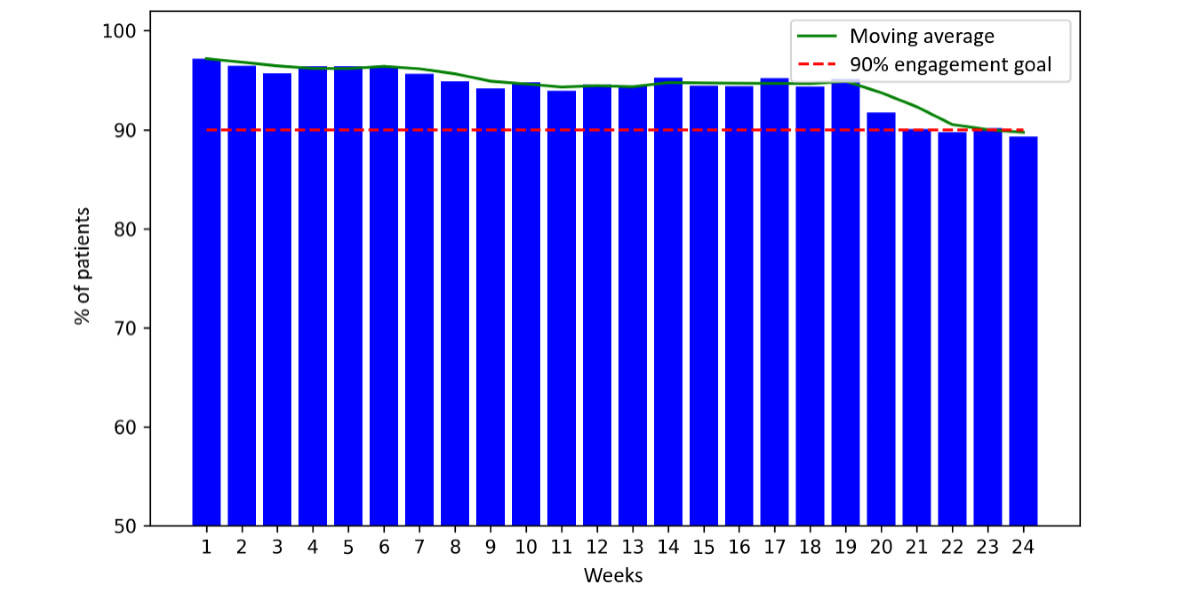
Percentage of active participants syncing their wearable device during the 24 weeks.

**Figure 7:**
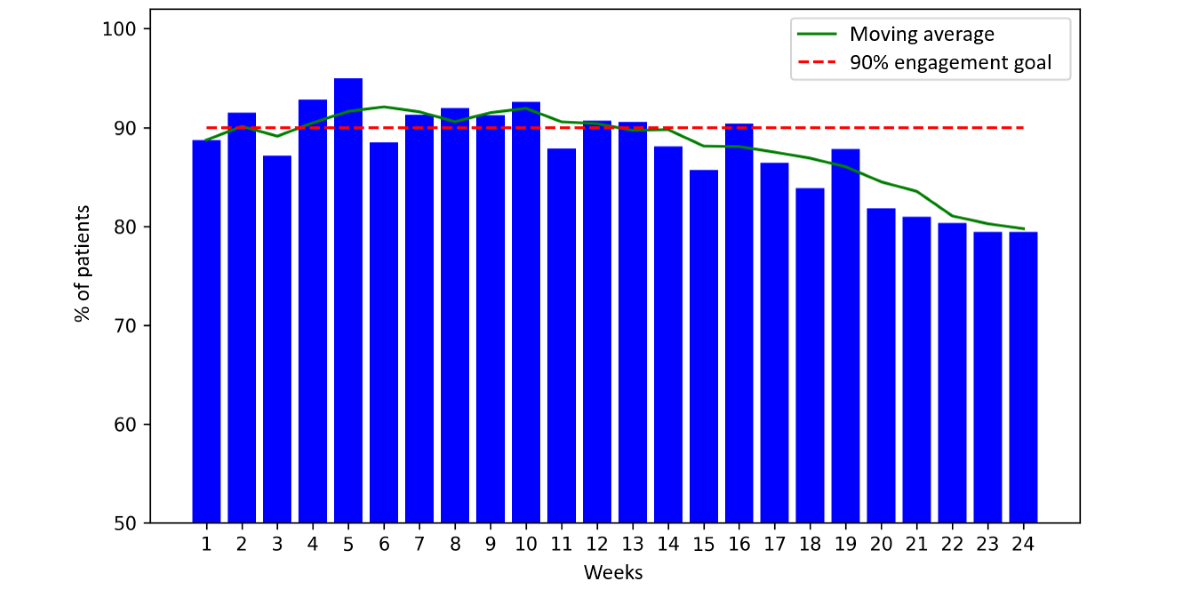
Percentage of active participants answering the questionnaire during the 24 weeks.

We assessed participant engagement based on the percentage of active participants completing the program tasks each week. Figures 5-7 show the weekly percentage of active patients measuring their BP, syncing their wearable device, and answering the questionnaire during the 24 weeks, respectively. We set an engagement goal of 90% for the study, which is represented by the red dashed lines in the figures. The average BP measurement engagement rate was 93% (SD 4.3%), and this rate was >90% for 19 (79%) out of 24 weeks. The average wearable syncing engagement rate was 94% (SD 2.4%), and this rate was >90% for 21 (88%) out of 24 weeks. The average questionnaire engagement rate was 88% (SD 4.9%), and this rate was >90% for 10 (42%) out of 24 weeks.

### Clinician Intervention

For the 128 participants completing 12 weeks in the study, an escalation notification was sent to the care team 8 times. There were 3.9% (5/128) unique patients who required manual outreach during the first 12 weeks. For the 102 patients completing 24 weeks in the study, an escalation notification was sent to the PHSO care team 11 times. There were 5.9% (6/102) unique patients who required manual outreach during the 24 weeks.

## Discussion

### Principal Findings

This study aims to assess the effectiveness of a fully digital, autonomous, and AI-based lifestyle coaching program in achieving BP control and high engagement among adults with hypertension. The key components of this program included detailed lifestyle data collection via both wearables and questionnaires and weekly lifestyle recommendations based on personalized, AI-based analytics delivered via a mobile app. The guidance supported the participant’s daily efforts to improve BP through behavioral changes that targeted physical activity, sleep hygiene, stress management, and dietary choices. Specifically, the program provided weekly guidance based on associations between lifestyle data and BP uncovered using ML and asked the participants to focus on the lifestyle factor with the greatest association. The precise lifestyle recommendations enabled participants to focus on the most relevant aspect of their lifestyle as opposed to receiving general guidance. Our intervention approach aligns with the Fogg Behavioral Model, which states that 3 elements (ability, motivation, and prompts) are essential for behavior change [37]. By directing participants to focus on 1 lifestyle behavior at a time, the intervention simplified compliance and therefore increased the ability of the participants to adhere to the recommendations. This targeted strategy likely bolstered participants’ motivation, as they could clearly see how specific lifestyle modifications directly influenced their BP. Each recommendation was delivered via a text message and prompted the user to take specific action. Furthermore, each recommendation was sent with a motivational message regarding their BP progress. We believe that this combination of personalized advice, ease of compliance, and motivational reinforcement contributed to our high engagement and improved BP outcomes.

We assessed multiple feasibility outcomes, including enrollment rate, adherence, and participant experience. In total, 59.9% (164/274) of the patients who initially expressed interest in joining the program ended up enrolling. Furthermore, although patients were recruited based on their last clinical BP reading, which required an SBP≥140 mm Hg or DBP≥90 mm Hg (stage-2 hypertension), many participants were not in the stage-2 range at baseline. Possible reasons for this include white coat hypertension [38] or that between the time of their last clinical BP reading and their enrollment in the study, they may have started taking BP medication or changed their diet. To improve the enrollment rate and ensure that patients who enroll have stage-2 hypertension, a new recruitment strategy is required. This new strategy could involve recruiting patients through PCP referrals. We hypothesize that this will increase the take-up rate due to increased trust from the more personal nature of the referral [39]. Furthermore, for the patients who are referred to the study, their PCPs would be instructed not to start the patients on any new BP medication or lifestyle intervention before the study, except in critical cases. This would help ensure patients joining the study are indeed in the stage-2 hypertension category. Another feasibility outcome we assessed was participant experience. While most participants (43/70, 61%) found the study tasks easy to incorporate into their daily routine, a few (3/70, 4%) found it difficult. These included difficulty in measuring BP due to work schedules and travel, caregiving responsibilities, and equipment and syncing issues. To address these challenges, the intervention should be more context aware and adapt the program tasks and recommendations based on patients’ circumstances. For example, a patient who works a night shift should not be asked to measure their BP at the same time or be given the same sleep recommendations as a patient who works during the day. Context-aware interventions would enhance the patient experience and increase the engagement rate.

Participants experienced a statistically significant decrease of 8.1 mm Hg and 5.1 mm Hg in SBP and DBP, respectively, after 24 weeks. Furthermore, this improvement was more pronounced in participants who started the program with stage-2 hypertension, achieving a 14.2 mm Hg and 8.1 mm Hg reduction in SBP and DBP, respectively. Reducing BP holds clinical significance not only for individuals with stage 2 hypertension but also for those with elevated BP or stage 1 hypertension. This is clinically meaningful as lower SBP values have been associated with progressively reduced risks of stroke, major cardiovascular events, and cardiovascular as well as all-cause mortalities [40]. In addition to BP improvement, the study demonstrates the intervention’s ability to maintain sustained engagement. However, the engagement rate dropped during the last 4 weeks potentially because the participants whose BP had improved through the program may have reduced their engagement as they did not feel the urgent need. In this study, the participant tasks remain consistent; however, participants may find it useful if the requirements are adaptive based on their health condition and preferences. It is worthwhile to design a dynamic mechanism that can adjust the extent and frequency of patient requirements based on the intervention progress. Both the BP and engagement results are achieved with minimal clinician intervention, primarily due to the autonomous nature of the intervention, demonstrating the potential scalability of this approach for hypertension management.

The observed BP improvement results from this study are comparable to those from clinician-led hypertension management programs [18-21]. The 3-month intervention program presented in the study by Wilson-Anumudu et al [18] combined lifestyle counseling with hypertension education, guided home BP monitoring, and support for taking medications and was led by either a registered nurse or certified diabetes care and education specialist. Patients with stage-2 hypertension who participated in this program experienced a 10.3 mm Hg and 6.5 mm Hg reduction in SBP and DBP, respectively, after 3 months. In the study by Milani et al [20], the 3-month digital intervention involved patients measuring their BP at least once per week and corresponding with pharmacists and health coaches to cocreate their treatment plan by choosing among various lifestyle modifications (eg, reducing dietary sodium) and medication options (eg, switching to generics or lower cost options). Patients with stage-2 hypertension participating in this program experienced a 14.0 mm Hg and 5.0 mm Hg reduction in SBP and DBP, respectively, after 3 months. Both interventions presented in the studies by Wilson-Anumudu et al [18] and Milani et al [20] assigned participants a designated hypertension coach who would provide lifestyle education and recommendations. These previous studies [18,20] primarily attribute their BP outcomes to the program’s support led by health professionals who interpreted BP data and supported lifestyle change. While health coach–based programs can produce meaningful BP improvements, the reliance on health coaches is highly time and resource intensive. Consequently, these approaches have limited scalability and accessibility as an individual health coach can only engage and care for a limited number of patients at a time. In contrast, our results demonstrate that a fully digital, AI-based lifestyle coaching program can produce clinically meaningful BP improvements comparable to those of programs led by health professionals. There is also potential for our approach to be used in conjunction with health coach–based programs. Under such a framework, our AI-based interactions and learnings from the patients can extend the reach of health coaches and provide them with more detailed insights about lifestyle factors impacting patients.

### Study Limitations and Future Directions

As this was a single-arm nonrandomized study, it was not possible to conduct a causal analysis due to the lack of a control group. In addition, regression to the mean is another limitation as participants with initially high BP values may naturally converge toward the average over time. Therefore, to conduct causal analysis and account for regression to the mean, a randomized controlled trial may be conducted to draw stronger conclusions in a future study. To gain additional insights into the effectiveness of the program, we can randomize patients into different treatment arms by providing different versions of the program. This could include varying the frequency or content of the lifestyle recommendations across the different treatment arms. Furthermore, we could investigate which lifestyle interventions, for example, increasing steps or improving sleep hygiene, result in greater BP improvements. With careful design, we can create a multiarm trial to investigate optimal engagement strategies and recommendations for different types of patients. Another limitation of this study is selection bias as the participants self-selected to enroll after receiving the recruitment flyer. To address this, we plan to recruit patients through PCP referrals. PCPs will refer their patients with high cardiovascular risk, who can benefit from our intervention. As previously mentioned, we hypothesize that this will increase the take-up rate due to increased trust from the more personal nature of the referral [39]. In addition, there is a need for a longer follow-up period as behavioral interventions can show improved outcomes during the first 6 months and then recidivism during the next 6 months. Finally, we did not collect socioeconomic data (eg, occupation, education, and income) from participants, preventing an analysis of how socioeconomic status impacts the program outcomes. In our future research, we will consider socioeconomic factors when analyzing the impact of the intervention. This analysis is imperative to ensure that the use of digital technologies does not contribute to an increased digital divide in health care and that all patients have equal access to high-quality health care [41,42].

### Conclusions

To address the challenges of poor patient engagement due to generic, nonpersonalized lifestyle guidance and limited scalability of care due to human coaching models, we propose an AI-driven, autonomous, precise lifestyle coaching program for patients with hypertension. Patients who enrolled in the program experienced a significant improvement in BP. The program maintained a high engagement rate with minimal intervention from the care team. As the burden of hypertension increases globally, the necessity to develop new strategies to achieve hypertension control at scale is greater than ever. An AI-based, autonomous approach to hypertension-related lifestyle coaching can increase scalability and accessibility to effective BP management, ultimately improving the cardiovascular health of our community.

## Appendix

Multimedia Appendix 1 Histogram showing the number of recommendations adhered to based on their difficulty rating. The average difficulty rating for recommendations that were followed was 1.97, indicating lower difficulty, whereas the average for those not followed was 3.67, indicating higher difficulty.

Multimedia Appendix 2 Participants’ responses to the study experience survey. This survey asked patients to rate the difficulty level of completing the study tasks, how useful they found the recommendations, and their experience using the app.

## Abbreviations

AI: artificial intelligence
API: Application Programming Interface
BP: blood pressure
DBP: diastolic blood pressure
ML: machine learning
PCP: primary care physician
PHSO: Population Health Services Organization
SBP: systolic blood pressure

## Data Availability

The data sets generated and analyzed during this study are not publicly available due to restrictions in the informed consent form.
