# Supplementary figures and images for: The Effect of an AI-Based, Autonomous, Digital Health Intervention Using Precise Lifestyle Guidance on Blood Pressure in Adults With Hypertension: Single-Arm Nonrandomized Trial
Source: JMIR Cardio. 2024 May 28;8:e51916. doi: 10.2196/51916 (PMC11167324; doi:10.2196/51916)

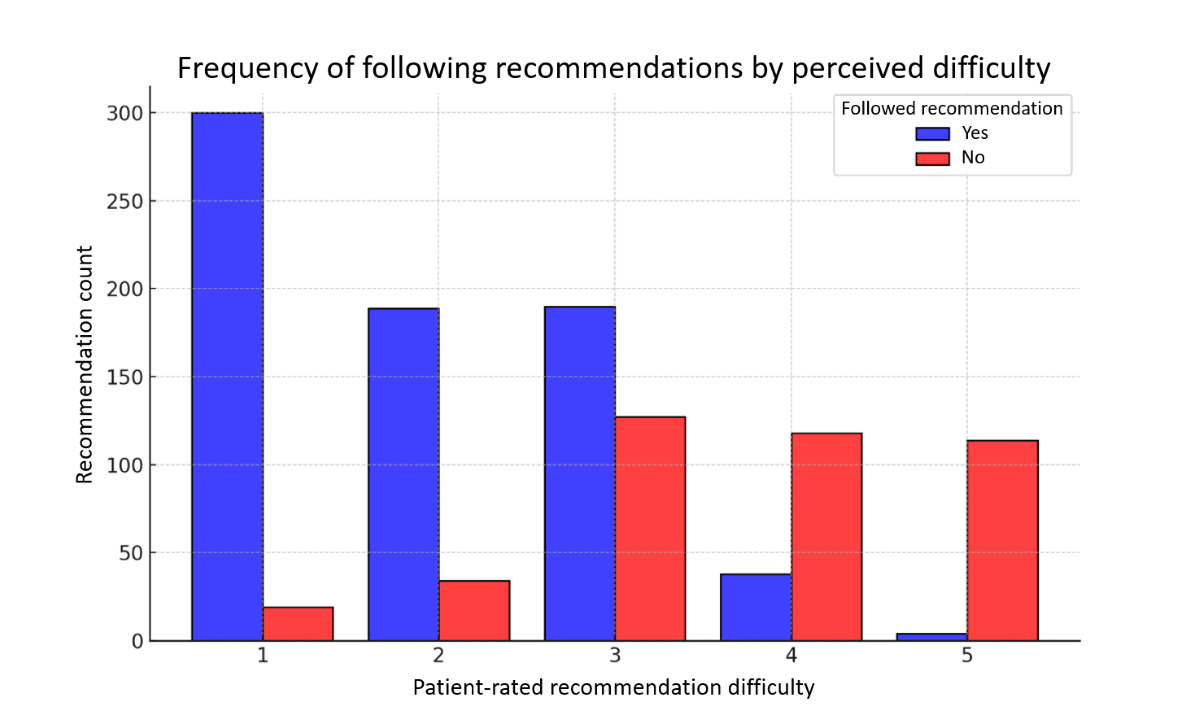

Supplement: Multimedia Appendix 1 [file cardio_v8i1e51916_app1.png]

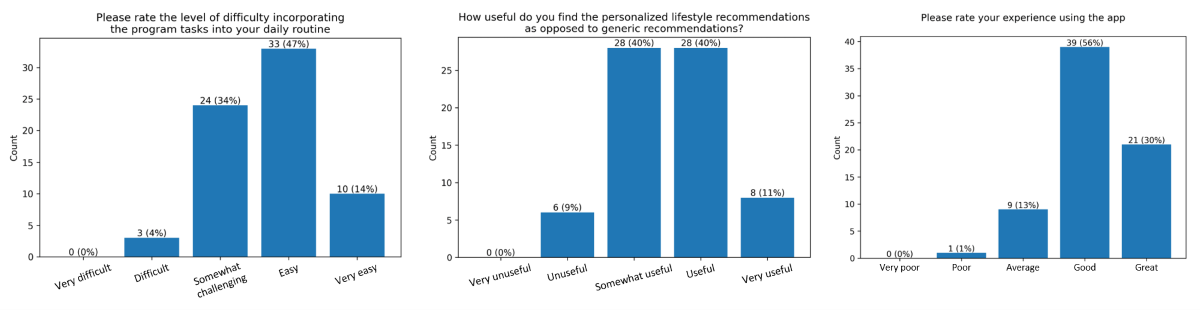

Supplement: Multimedia Appendix 2 [file cardio_v8i1e51916_app2.png]
